# The origins of malaria: there are more things in heaven and earth …

**DOI:** 10.1017/S0031182014000766

**Published:** 2014-06-25

**Authors:** P. J. KEELING, J. C. RAYNER

**Affiliations:** 1 Department of Botany, Canadian Institute for Advanced Research, Evolutionary Biology Program, University of British Columbia, Vancouver, BC V6T 1Z4, Canada; 2 Malaria Programme, Wellcome Trust Sanger Institute, Wellcome Trust Genome Campus, Hinxton, Cambridge CB10 1SA, UK

**Keywords:** Apicomplexa, *Plasmodium*, evolution, origin, phylogeny

## Abstract

Malaria remains one of the most significant global public health burdens, with nearly half of the world's population at risk of infection. Malaria is not however a monolithic disease – it can be caused by multiple different parasite species of the *Plasmodium* genus, each of which can induce different symptoms and pathology, and which pose quite different challenges for control. Furthermore, malaria is in no way restricted to humans. There are *Plasmodium* species that have adapted to infect most warm-blooded vertebrate species, and the genus as a whole is both highly successful and highly diverse. How, where and when human malaria parasites originated from within this diversity has long been a subject of fascination and sometimes also controversy. The past decade has seen the publication of a number of important discoveries about malaria parasite origins, all based on the application of molecular diagnostic tools to new sources of samples. This review summarizes some of those recent discoveries and discusses their implication for our current understanding of the origin and evolution of the *Plasmodium* genus. The nature of these discoveries and the manner in which they are made are then used to lay out a series of opportunities and challenges for the next wave of parasite hunters.

## A NEW AGE OF PARASITE DISCOVERY

Scientists in general, and perhaps parasitologists in particular, have always been driven by a need to define the landscape in which they work. Almost as soon as Charles Laveran first glimpsed the wink of a hemozoin crystal down his microscope there was a rush to categorize these strange new organisms – to give labels to the phases of their extraordinarily complex life cycle, and to group them into species. After a period of occasionally fractious dispute, with the distinction between *Plasmodium vivax* and *Plasmodium ovale* proving the most difficult to resolve, by 1922 the four major human *Plasmodium* species had been defined and named. Discovery of new *Plasmodium* species in other hosts continued for some time, with a particularly golden era of discovery in Asian monkeys in the early 1960s. However by the late 1960s some of the world's most eminent malariologists felt that they had a secure enough grip on the *Plasmodium* genus that they could define it in elegant and definitive textbooks (Garnham, 1966; Coatney *et al.* 1971), complete with the beautifully detailed illustrations of each species that are still widely used in teaching and research presentations to this day. At the same time, ultrastructure and molecular phylogeny combined to give us an equally well-defined view of the broader place of *Plasmodium* in the tree of life, and amongst its close relatives in the phylum Apicomplexa.

In the past 5 years, however, the clear and definitive worldview described in these textbooks has been radically overhauled by successive waves of new discoveries, powered by a combination of molecular diagnostic technologies and extensive sampling efforts. Some of these searches involve painstaking observations of blood smears from wild-caught animals and would be instantly recognizable to the original *Plasmodium* pioneers. Others would appear completely alien, involving ape faeces collected off forest floors in West Africa and massive molecular surveys of the world's greatest coral reef ecosystems. Together these studies have forced a re-evaluation of the origin of the most deadly human malaria species, *Plasmodium falciparum*, the source of the rodent malaria parasite species and even the very origins of the phylum Apicomplexa itself.

## UNEXPECTED ROLES FOR CORAL AND PHOTOSYNTHESIS IN APICOMPLEXAN EVOLUTION

Tracing the evolutionary history from *Plasmodium* back through its apicomplexan relatives and beyond, there was a point at which the parasitic lifestyle that characterizes the entire apicomplexan lineage originated. Finding this point in time and explaining how and why this massive transition took place have long been difficult questions. Over a decade ago the answers took an unexpected turn with the discovery that *Plasmodium* and other apicomplexans contained a plastid (generally called the ‘apicoplast’; McFadden *et al.* 1996; Wilson *et al.* 1996; Kohler *et al.* 1997), an organelle usually used by plants and algae for photosynthesis. So the question became more precise, but stranger: how did a presumably photosynthetic ancestor turn into an obligate intracellular parasite of animals? Recently, thanks to some good old-fashioned organism hunting, these two questions are merging into a new way to look at the deep origins of apicomplexans.

**Fig. 1.**
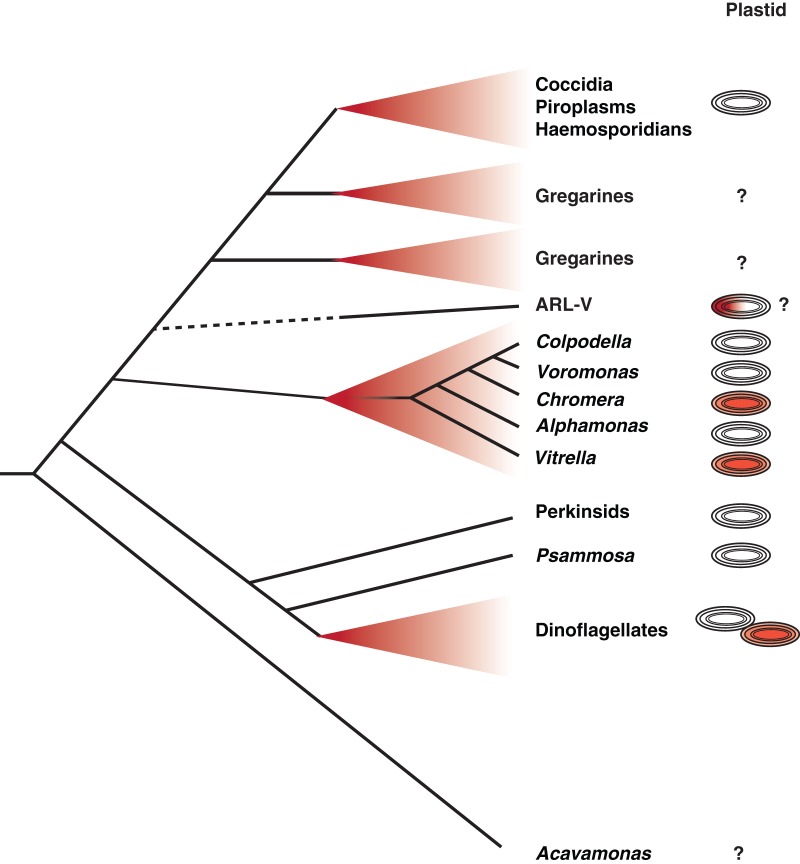
Schematic representation of relationships between apicomplexan parasites and their closest relatives and the evolution of their plastids. The closest known branch to the ‘true’ apicomplexans (at top, including Coccidia, Piroplasms, Haemosporidians and the paraphyletic Gregarines) is a biologically undescribed lineage known only from plastid environmental surveys, the so-called ARL-V lineage. The nearest relatives that have been biologically characterized include a diverse array of predatory flagellates (*Colpodella, Voromonas* and *Alphamonas*), photosynthetic coral symbionts (*Chromera* and *Vitrella*) and a large number of unknown environmental lineages (many from coral, but also many from other environments). These are all in turn related to a large group including dinoflagellates and their closest relatives, the Perkinsids and *Psammosa*, both of which possess structures homologous to the apical complex, and the enigmatic predator *Acavamons*. The column to the right summarizes what we know about plastids in each lineage: red plastids indicate photosynthesis, colourless plastids indicates plastids that are known but non-photosynthetic. ARL-V is hypothesized to be photosynthetic but this has not been tested, and dinoflagellates contain about 50% photosynthetic and non-photosynthetic species. Lineages for which no plastid has been detected are indicated by a question mark.

Critical to this new understanding was the discovery of living descendants of a recent ancestor of the apicomplexans – in effect photosynthetic members of the apicomplexan lineage (Moore *et al.* 2008). *Chromera* and *Vitrella* are two new genera of fully photosynthetic algae that were isolated from coral reefs, and branch near the base of the phylum Apicomplexa in molecular phylogenetic trees (Moore *et al.* 2008; Janouskovec *et al.* 2010) (Fig. 1). The plastid genomes of both have been fully sequenced, and demonstrated that apicomplexan plastids are derived from the same red algal endosymbiont that also gave rise to plastids of dinoflagellate and stramenopile algae (Janouskovec *et al.* 2010). The *Chromera* and *Vitrella* plastid sequence data also revealed another unexpected finding. When they were compared with bacterial environmental sequencing data, it become apparent that environmental microbial populations are heavily contaminated with sequences from eukaryotic plastid genomes, presumably derived from cyanobacteria. Comparing these inadvertent but vast surveys of plastid sequences to known plastid genomes revealed that *Chromera* and *Vitrella* are just the tip of the iceberg: there is a brace of new and unknown plastid lineages, all clustering at the base of the apicomplexans. Remarkably, all of these are specifically associated with coral reef environments: coral samples consistently contain sequences from apicomplexan-related plastids, and a variety of other environments, many of which are far more thoroughly sampled, do not. Some of these are related to *Chromera* and *Vitrella*, most are new and independent lineages (Janouskovec *et al*. 2012, 2013). Indeed, the most common apicomplexan relative from coral is a new lineage known only as Apicomplexan Related Lineage-5 (ARL-V: Fig. 1). ARL-V is the closest known relative of apicomplexans, but its biology is totally unknown: it is as yet defined only by DNA sequences (Janouskovec *et al*. 2012, 2013).

Coral has never been regarded as a particularly important habitat for apicomplexans (only one as yet unidentified coral parasite known as Genotype N has been investigated at all), but these new data suggest something much more – that coral may be the cradle of apicomplexan origins (Toller *et al.* 2002). It is possible that the association between the ancestor of apicomplexans and animals began as a mutually beneficial one based on photosynthesis with an ancient and likely now extinct lineage of corals, similar to the association between modern corals and zooxanthellae. Later this association soured and became one-sided, perhaps when the lineage leading to apicomplexans lost photosynthesis but retained their ability to invade coral cells. This would tip the balance to an association more like the parasitic ones we see today. This story sounds appealing, and may even be partly true, but the truth is almost certainly more complex. For a start, another lineage is known to branch at the base of apicomplexans, and its members are neither parasitic nor photosynthetic. Instead, the colpodellids are free-living heterotrophs that seem to specialize in attacking and eating other eukaryotes using a feeding apparatus homologous to the apical complex (Kuvardina *et al.* 2002; Leander and Keeling, 2003). The exact relationship between colpodellids, *Chromera, Vitrella* and apicomplexans remains unclear, so it is too early to make firm conclusions about which, if either, kind of lineage made the transition to obligate parasitism. However, the nearly perfect correlation between the photosynthetic members of the lineage and corals gives us a number of new intriguing leads to follow (Janouskovec *et al*. 2010, 2012, 2013). Whether these resolve the ultimate origin of apicomplexan parasitism remains to be seen, but at the very least they have provided us with a completely new context with which to examine the question and new perspectives from which to view the parasites.

## FINDING CONNECTIONS IN UNEXPECTED PLACES – bat *PLASMODIUM* REVEAL NEW INSIGHTS INTO THE ORIGINS OF RODENT MALARIA

Just as surveys of coral reefs have revolutionized our understanding of the origins of the phylum Apicomplexa, an even more recent survey of apicomplexan parasites of bats has begun a new revolution, this time in our understanding of the origins of rodent parasites. *Plasmodium* parasites infecting rodents were first observed in 1948 by two Belgian scientists, Vincke and Lips, in what is now the Democratic Republic of Congo (Vincke and Lips, 1948). This initial description, of *Plasmodium* sporozoites in an infected mosquito, led to a series of expeditions that defined a number of *Plasmodium* species that infected African thicket rats (primarily *Grammomys* and *Thamnomys* species). Four species, *Plasmodium berghei, Plasmodium yoelii, Plasmodium chabaudi* and *Plasmodium vinckei* were all transferred to laboratory mice, where they have been extraordinarily useful tools for understanding malaria biology. These rodent species are non-infectious to humans, which makes working with them in a laboratory setting straightforward. While they have proven controversial models for specific aspects of malaria pathology (Craig *et al.* 2012), there is no doubt that they also offer many completely unique advantages, and have enabled experiments and approaches that would simply not have been possible without them. Most notably, rodent *Plasmodium* species have allowed systematic analysis of liver and mosquito stages, which are technically demanding or even inaccessible when working with human parasites (Lindner *et al.* 2012), and are proving amenable to high-throughput experimental genetics and systematic immunology. While transferring findings from rodent to human *Plasmodium* will always be critical for validation, and for certain questions such as studying host-parasite interactions which may evolve rapidly between species, in general these African thicket rat parasites have proven invaluable for studies of basic *Plasmodium* metabolism and biology.

**Fig. 2.**
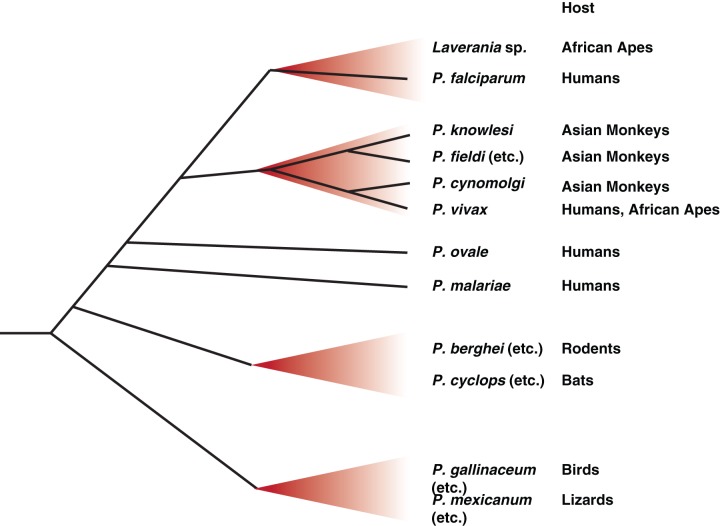
Schematic representation of the major radiations amongst *Plasmodium* species. Since becoming parasites of vertebrates, the genus *Plasmodium* has expanded to infect a wide variety of hosts. Only a handful of these species, those referred to specifically in the text as well as some other major groupings, are represented here. The precise relationships between the species are not always known, so branch positioning is indicative rather than definitive – more detailed analyses are available elsewhere (Martinsen and Perkins, 2013). Human parasites originate from *Plasmodium* clades that have expanded in related groups of hosts, including the *Laverania* radiation in African apes, which includes the most deadly form of human malaria, *P. falciparum*, and an expansion of *P. vivax-*related parasites in African apes and Southeast Asian monkeys. The other two major human malaria parasites, *P. ovale* and *P. malariae*, also have relatives in African apes, but the full diversity and relationship between these species is not currently known. Recent works indicates that rodent and bat *Plasmodium* parasites are closely related, with possible host switching occurring on more than one occasion (Schaer *et al.* 2013).

However, while they may have proven an experimental boon, where these rodent parasites fit in the overall picture of *Plasmodium* phylogeny has always been somewhat of an anomaly, lying clearly outside the primate *Plasmodium* radiation that includes human parasites, but with no other close relatives (Escalante *et al.* 1998). This is largely because only a very few rodent *Plasmodium* samples exist – no new rodent *Plasmodium* isolates have been obtained since those initial expeditions in the 1940s and 50s, although samples from these initial expeditions have recently again become available to researchers through a new repository, from which new species may emerge (http://www.malariaresearch.eu). It has taken a new expedition in West Africa, similar in pioneering spirit to those performed more than 50 years ago, to provide some context. Surveying bats in remote forests of Guinea, Liberia and Cote d'Ivoire revealed multiple haemosporidian parasites, including two *Plasmodium* species (Schaer *et al.* 2013). These species, *Plasmodium voltaicum* and *Plasmodium cyclopsi*, had been previously identified but classified only based on morphology. Molecular phylogeny of the new samples, using a combination of mitochondrial, apicoplast and nuclear genes, revealed the surprising finding that these bat parasites fall within the rodent *Plasmodium* clade (summarized in Fig. 2), despite the distant evolutionary relationship between their mammalian hosts (Schaer *et al.* 2013). Host switching in *Plasmodium* species is well-established through work in avian parasites (Ricklefs and Fallon, 2002; Ricklefs *et al.* 2004; Beadell *et al.* 2009), although it has not been as frequently described for mammalian species. In this case, switching may be facilitated by the fact that African thicket rats are arboreal, and therefore are presumably exposed to the same *Anopheles* vectors as bat species. Further work is clearly needed, including the exciting prospect of attempting to transfer some of these bat *Plasmodium* species to laboratory rodents, but again this discovery emphasizes that systematic screening of a wide range of biological specimens, an approach that had fallen somewhat out of favour in the *Plasmodium* field, can yield new and completely unexpected insights.

## FAECAL SAMPLES LEAD TO A NEW UNDERSTANDING OF THE ORIGINS OF *P. FALCIPARUM*

Perhaps the most widely reported example of this new wave of *Plasmodium* discovery lies in a series of papers investigating the origins of *P. falciparum*, the species that causes almost all human malaria mortality. From the very earliest days of *Plasmodium* discovery it was established that African apes were naturally infected with *Plasmodium reichenowi*, a parasite that was morphologically nearly identical to human *P. falciparum*, but appeared to be a distinct species (Reichenow, 1920). Only a single isolate of *P. reichenowi* was ever obtained for study, from a wild chimpanzee that had been transported to the USA in the 1950s (Collins *et al.* 1986). There the matter rested for decades, with *P. falciparum* and *P. reichenowi* thought to be sister species completely isolated from all other known *Plasmodium*. In the past five years this view has been comprehensively overhauled by a variety of means (Rayner *et al.* 2011), including harnessing the power of modern molecular techniques to study samples that our parasite-prospecting forebears would never have dreamed of – ape faeces. Such unusual samples were required because unlike bats, and even more so than coral reefs, wild-living African apes are highly protected, and invasive capture and collection approaches for wild primates are clearly inconceivable. However, ape blood is available in very restricted circumstances from samples taken for health surveillance of captive animals, and in 2009 a study of samples from two pet chimpanzees revealed what appeared to be a new species of *P. falciparum*-related parasite, *Plasmodium gaboni* (Ollomo *et al.* 2009). A series of similar studies using small numbers of samples from captive apes followed, with somewhat contradictory results or interpretation, although all agreed that there appeared to be a much greater diversity of *P. falciparum-*related parasites in great apes than was previously realized, and potentially more than could be defined as a single species (Rich *et al.* 2009; Duval *et al.* 2010; Prugnolle *et al.* 2010).

It was the discovery that *Plasmodium* DNA could be amplified from faecal samples (Prugnolle *et al.* 2010), which can be non-invasively collected in large numbers from multiple sites, that generated a sufficient sample size to clarify the matter, just as similar studies of HIV-related virus RNA from ape faecal samples had clarified the origin of human HIV (Keele *et al.* 2006). A survey of nearly 3000 such samples revealed six clades of *P. falciparum-*related parasites, now collectively referred to as sub-genus *Laverania* (Liu *et al.* 2010). All six *Laverania* clades appeared to be host-species specific, at least in wild-living animals, with some species only infecting chimpanzees and others only infecting gorillas, even when the ape species are sympatric. Most significantly all human *P. falciparum* sequences cluster within the radiation of a gorilla-specific parasite, implying that the human *P. falciparum* epidemic is the result of a single transmission event from this gorilla parasite species, which is now referred to as *Plasmodium praefalciparum*. While the definition of these *P. falciparum-*related clades as individual species might not fit classical definitions due to the current lack of morphological data, the impossibility of obtaining blood samples from wild-living apes, coupled with the frequent occurrence of mixed infections within the same animal will make this more rigorous naming barrier a hard, if not impossible, hurdle to clear. However, these studies do clearly show that *P. falciparum* is not the orphan that it was once thought to be, but, much like the rodent *Plasmodium* species, it nestles within a much broader radiation of related species in related hosts, with host switching occurring in some branches and not others (summarized in Fig. 2).

## MOVING FROM DIAGNOSTICS TO DEFINITIONS – the power of genomes

These three discoveries are far from the only examples of recent changes to our understanding of how and where apicomplexan parasites come from – the establishment of frequent zoonotic transmission of *Plasmodium knowlesi* from macaques to humans in Malaysia (Singh *et al.* 2004), or the subdivision of *P. ovale* into two sub-species (Sutherland *et al.* 2010) both stand as further evidence that we are in the process of a radical realignment of *Plasmodium* phylogeny. Why, decades after the initial age of *Plasmodium* discovery, is this occurring? Part of the answer is simply that we are now looking in new places – all of the studies discussed in this review rely on surveying samples that would not have previously been considered for systematic study, either because of logistical difficulties in obtaining them (such as trapping large numbers of bats), or because they were not previously realized to contain useable information (such as ape faeces or samples from coral reefs). However, a critical part of the answer, as with most scientific breakthroughs, lies in advances in technology – in this case the systematic application of DNA sequencing. The first wave of parasite hunters could only define what they saw based on microscopy and morphological characteristics. The new wave, led by researchers studying avian malaria where samples have always been limiting, can use highly sensitive PCR for detection, an array of related genome sequences to provide potential targets for amplification, and, in the case of the origins of Apicomplexa, massive molecular surveys of whole microbial ecosystems from which to pluck sequences.

The success of these approaches means that the question of morphology has in fact become somewhat a controversial one, with some researchers questioning of the validity of species identification based entirely on molecular means and emphasizing that morphology will always play a critical role in defining apicomplexan species (Perkins *et al.* 2011; Valkiunas *et al.* 2011). Although there may be differences of emphasis, most researchers would agree that microscopy and morphology will always play a key part in our understanding of *Plasmodium* and Apicomplexan species and should remain the gold standard for species identification (Perkins, 2014), just as microscopy remains the gold standard for clinical diagnosis. However, there is equally no doubt that in some circumstances, such as the case of African ape *Plasmodium* species, or the coral-dwelling ARL-V, morphological definitions will be slow if not impossible to perform. Samples from wild-living apes, for example, will quite rightly never be possible to obtain because of ethical restrictions, and while opportunistic investigation of samples taken for health-related reasons from captive apes is sometimes possible, the frequency of multiple infections with different *Laverania* species will make interpretation difficult in most circumstances. For the more deep ancestors of apicomplexans, it may simply never be possible to obtain samples from which morphological identification can be performed. Until such a time that morphological data become available, if ever, the scientific community clearly needs some sort of framework to discuss findings and definitions, and that framework currently relies on molecular phylogeny. While such an approach might be less palatable to some, it is far from a unique approach – research in bacterial ecology, for example, has been coping with large numbers of unidentified ‘molecular taxa’ for over a decade.

While molecular diagnostics are clearly here to stay, and in some cases may never be replaced by other diagnostic methods, it is critical that for these new species we move beyond simple diagnosis to clear definition. An important element of this will be the generation of complete genome sequences for all these new parasites. Not many years ago that would have seemed a very distant prospect, but the advent of next-generation sequencing technology means that we should now expect fairly rapid progress towards full genomic definitions. In some cases, where samples are limited or are of limited quality, generating complete genomes will be challenging even using these new technologies. However, these challenges are not radically different to those facing environmental microbiologists, who often must deal with uncultivated organisms, or population genetic studies of human *Plasmodium* species, where the drive is to extract the maximum genomic information from the smallest possible volume of clinical samples such as dried blood spots. Developments in the field of human *Plasmodium* population genetics, such as methods to digest human DNA and leave *Plasmodium* DNA untouched (Oyola *et al.* 2013), or hybrid capture approaches to pull out *Plasmodium* material from mixed samples (Melnikov *et al.* 2011), will clearly be applicable to even the most challenging samples such as small volumes of bat blood.

Once genomes of these new species are generated, comparative genomics will hopefully lead to specific hypotheses that can be tested experimentally. In the case of the *Laverania*, genomes exist for only two members of the sub-genus, *P. falciparum* and *P. reichenowi* (Otto, Newbold & Berriman, manuscript submitted), and the as yet unreported genome of *P. praefalciparum* will clearly be of great interest in understanding the origin of human *P. falciparum* and of severe malaria pathogenesis. Comparative genomics will also enlighten one of the most interesting aspects of *Laverania* parasites, their apparent strict host restriction. Recent *in vitro* studies suggest that in the case of *P. falciparum*, host specificity may be due at least in part to specificity in a critical protein-protein interaction that mediates erythrocyte invasion (Wanaguru *et al.* 2013). Complete genomes from multiple *Laverania* species will allow a much more comprehensive test of this hypothesis. Similarly, genomes of *P. voltaicum* and *P. cyclopsi* will be of great interest to compare with the genomes of rodent parasites. The fact that the entire *Plasmodium* life cycle can be replicated in these rodent models will allow for a much more systematic analysis of the factors that control switching between bat and rodent hosts, including analysis of mosquito and liver stages. As for the deeper evolutionary origin of apicomplexans as a whole, complete genome sequences for both the cultured relatives, *Chromera* and *Vitrella*, is a relatively straightforward problem, and both are currently underway. These genomes should allow some insights into a number of interesting questions about the ancient transition from free-living phototroph to obligate parasite, and perhaps even generate more specific hypotheses about the possible role of coral in the origin of parasitism. However, a major piece of the puzzle for understanding the origin of apicomplexans remains the uncultured, unidentified and generally unknown lineages, especially ARL-V, which is the closest relative of apicomplexans that we currently know of, but which has never even been seen under the microscope as yet.

## CONCLUSIONS AND A WAY FORWARD: THE NEED FOR SYSTEMATICS AND AGREEMENT

There is no question that many new discoveries await, particularly as genome sequencing becomes applicable to ever smaller and more complex samples. In this concluding section, we highlight a series of challenges that have sometimes bedevilled the field in the past, and suggest ways in which the community can move forward in a more systematic and rigorous manner.

### Discovery

The simple message here is that new sample surveys are urgently needed, and that no sample should be overlooked. What is missing in the current surveys? In terms of the broader context of apicomplexan origins, the link between coral reefs and photosynthetic relatives of apicomplexans is helpful, but our understanding of these coral communities remains rudimentary. Moreover, we have not even begun to sample the diversity of non-photosynthetic apicomplexan relatives, colpodellid predators, which inhabit a range of environments, so we should not be restricted to focusing on coral. In terms of *Plasmodium* specifically, there is clearly much more to discover in African apes, with *P. vivax, P. ovale* and *Plasmodium malariae*-related parasites all detected, but their relationship to the human counterparts not yet understood (Duval *et al.* 2009; Hayakawa *et al.* 2009; Prugnolle *et al.* 2013). A systematic revisiting of *Plasmodium* species in Asian monkeys is also long overdue, especially because type specimens of a number of species, including *Plasmodium cynomolgi, Plasmodium fieldi, P. knowlesi, Plasmodium coatneyi* and *Plasmodium simiovale* now exist in public repositories. New World monkeys also represent a relatively untapped field. *Plasmodium vivax* and *P. malariae-*related species have been identified in multiple New World monkey species, and are widely assumed to be anthroponotic (Tazi and Ayala, 2011), but the range of host species for these simian parasites, and how far they are diverging in these new hosts, is not known. The list of fascinating specimens is long, with reptile *Plasmodium* a particularly untapped field. Many exciting discoveries await.

### Diagnostics

It is clear that several *Plasmodium* species frequently co-exist in the same hosts. It will therefore be important to develop molecular diagnostic approaches that are both broad and deep to ensure that rarer co-infecting species are not missed. An excellent example is non-*Laverania* parasites in African apes. *Plasmodium vivax, P. malariae* and *P. ovale*-related species clearly exist in apes, but relatively few sequences have been generated so far when compared with *Laverania* sequences. This is likely to be because the prevalence, and presumably parasitaemia, of *Laverania* parasites is so high that it swamps the signal from non-*Laverania* parasites when broad pan-*Plasmodium* primers are used for screening, in much the same way that *P. ovale* and *P. malariae* in Africa are likely under-estimated because they occur at much lower densities than the dominant *P. falciparum*. It will therefore be essential to use both broad genus spanning and targeted species-specific approaches for screening, and resurveying the same sample set with targeted primers once the extent of diversity is established with broad primers will be an important approach.

### Standardization

There is an urgent need for consistency in which gene fragments are used for diagnosis in order to maximize comparison between studies. While there are some general standards in the field, primers and specific gene coordinates vary widely between studies and even the genome being targeted can differ – for *Plasmodium* species mitochondrial gene fragments are widely used, while for coral-dwelling apicomplexan relatives plastid sequences are used, and for apicomplexan relatives in other environments nuclear sequences are used. Agreement on such matters is never easy, but where it can be made the results are incredibly powerful. The most striking examples are DNA diversity barcodes now used by multiple scientific communities such as the iBol community attempting to catalogue all multicellular eukaryotes (ibol.org), and the protist barcode group who are using a two-tiered barcoding approach (Pawlowski *et al.* 2012). In both of these cases large research communities have agreed to co-ordinate efforts and standardize markers, and in doing so have allowed the systematic identification and comparison of literally thousands of species. The *Plasmodium* and apicomplexan communities would do well to follow suit and agree on standard gene fragments for amplification wherever possible.

### Definition

As noted above, while diagnosis is important and useful, it needs to be followed rapidly by definition, which means whole genome sequences. Rapid advances in sequencing technology, coupled with methods to increase sample quantity (for example by whole genome amplification) and decrease contamination from non-*Plasmodium* sequences (by hybrid selection, or digestion of non-target sequences) offer hope that we can rapidly move to complete genomes from even small and difficult samples. In parallel, advances in single cell genomics offer even greater promise. The field is moving towards the ability to physically isolate a single parasite and generate data from the whole genome or whole transcriptome, which will quickly open newly identified species to functional analysis, as well as rapid population genomics analysis. The advantage of genomic definition is that there is no need for agreement on specific fragments to focus on, and the principles of open data access and sharing are deeply embedded in the genomics community, which will facilitate comparisons between studies.

### Taxonomy fit for purpose

By pushing our sampling and identification efforts to the limits of new technology, we will inevitably surpass current constraints of taxonomic regulations. Specifically, this means finding ways to deal with new taxonomic units that are defined in non-traditional ways. For example, it is now conceivable to have complete genomes from organisms we have never seen, but these cannot be formally ‘described’. Indeed, some journals refuse to allow descriptions even with comprehensive microscopy, if the organism is not available in culture. Luckily this is not a new problem, and we can examine how bacterial ecology has dealt with the taxonomic turmoil created by metagenomics and environmental tag sampling for some hints as to how we might proceed.

### Caution

As we discover more and more species, as a community we need to set our bar higher for what actually constitutes a discovery. In recent years there have been publications that have taken even a single sample of a new species or a new host as the only proof required (Prugnolle *et al*. 2011*a*, *b*; Sharp *et al*. 2011). While discoveries based on small sample numbers can be later borne out, such as the initial discovery of *P. gaboni* based on only two samples, it is also true that large-scale surveys of multiple samples using highly sensitive molecular diagnostics present very real risks of sample mix up and contamination, even in the most careful labs. Indeed, as genome sequencing has scaled up, so too has the problem of cross contamination, so now low-level contaminants appear in many or all large-scale sequence projects. In studies aiming to produce fully assembled genomes this is not a great problem, but in environmental surveys seeking rare species it can be a very serious problem indeed. Independent validation and caution in interpretation are scientific watchwords, but should be doubly respected where discovery of new species is concerned.

### Nomenclature

When multiple groups are working in the same scientific area, differences in nomenclature inevitably arise. While these are unavoidable to some extent, respect for the scientific principle of deferring to the first names given to a new species would go a long way to resolving some of the conflicts (just as the same principle would be helpful for *Plasmodium* gene and protein names). When the literature becomes too entangled, as a field we should consider the old-fashioned approach of getting the relevant people in the same room until a consistent nomenclature can be agreed upon. Consistency in naming will only help drive the field forward.

These are exciting times for those of us working on the evolution of and new species discovery in apicomplexans, and the combination of new sample collections, molecular diagnostics and new genomic technologies make it likely that more breakthroughs will follow. By using consistent and sensitive approaches, applying them to every sample set that can be collected, and adhering to the most rigorous principles of scientific proof, the breakthroughs of the last few years are likely to be only the tip of the iceberg.
